# Клиническая и молекулярно-генетическая характеристика серии случаев синдрома Флоатинг-Харбор

**DOI:** 10.14341/probl13530

**Published:** 2025-07-22

**Authors:** Н. А. Макрецкая, О. Р. Исмагилова, Е. А. Шестопалова, М. В. Шарова, О. А. Левченко, А. В. Болмасова, М. В. Булах, В. В. Забненкова, А. А. Орлова, А. А. Колодкина, О. П. Рыжкова, А. В. Поляков, А. Н. Тюльпаков

**Affiliations:** Медико-генетический научный центр им. акад. Н.П. Бочкова; Медико-генетический научный центр им. акад. Н.П. Бочкова; Медико-генетический научный центр им. акад. Н.П. Бочкова; Медико-генетический научный центр им. акад. Н.П. Бочкова; Медико-генетический научный центр им. акад. Н.П. Бочкова; Медико-генетический научный центр им. акад. Н.П. Бочкова; Медико-генетический научный центр им. акад. Н.П. Бочкова; Медико-генетический научный центр им. акад. Н.П. Бочкова; Медико-генетический научный центр им. акад. Н.П. Бочкова; Национальный медицинский исследовательский центр эндокринологии им. академика И.И. Дедова; Медико-генетический научный центр им. акад. Н.П. Бочкова; Медико-генетический научный центр им. акад. Н.П. Бочкова; Медико-генетический научный центр им. акад. Н.П. Бочкова; Российская детская клиническая больница

**Keywords:** низкорослость, лицевой дисморфизм, синдром Флоатинг-Харбор, SRCAP

## Abstract

Синдром Флоатинг-Харбор — аутосомно-доминантное заболевание, входящее в группу наследственных вариантов низкорослости. Клиническая картина заболевания включает в себя задержку роста, речевого развития и специфические лицевые аномалии: лицо треугольное с узким подбородком, глаза глубоко посаженные, фильтр короткий, макростомия, тонкая верхняя губа, нос с узкой переносицей и широкими основанием и кончиком, свисающая колумелла. Формирование синдрома ассоциировано с гетерозиготными патогенными вариантами в 33 и 34 экзонах гена SRCAP, приводящие к укорочению длины белка. Гетерогенность и отсутствие специфических клинических проявлений зачастую усложняет постановку данного диагноза, чем обусловлена необходимость в молекулярно-генетической верификации. В настоящей работе нами представлено первое в Российской Федерации описание 6 пациентов с доказанным синдромом Флоатинг-Харбор.

## Abstract (en)

Floating-Harbor syndrome is an autosomal dominant genetic disorder that is part of a group of growth retardation. The main clinical features of syndrome are short stature, delayed speech development and peculiar facial features: triangular face with a narrow chin, deeply set eyes, a short filter, a macrostomy, a nose with a narrow bridge and wide base and tip, a drooping columella. The syndrome is associated with heterozygous pathogenic variants were truncating and occurred in 33 and 34 exons of SRCAP. Heterogeneity and the absence of specific clinical features complicates the formulation of this diagnosis, which necessitates molecular genetic verification. In this paper, we present the first description of 6 patients with proven Floating-Harbor syndrome in the Russian Federation.

## АКТУАЛЬНОСТЬ

Синдром Флоатинг-Харбор (СФХ) (OMIM #136140) — редкое аутосомно-доминантное заболевание, характеризующееся выраженным отставанием роста, задержкой речевого развития, скелетными аномалиями и лицевыми дисморфиями: треугольное лицо с узким подбородком, глубоко посаженные глаза, короткий фильтр, макростомия, тонкая верхняя губа, нос с узкой переносицей и широкими основанием и кончиком, выступающая колумелла [1]. На сегодняшний день в мировой литературе описано более 100 клинических случаев СФХ [1–5]. Несмотря на то, что клиническая картина заболевания хорошо изучена, большинство проявлений, в частности низкорослость и особенности лица, не являются специфическими. Дифференциальная диагностика в данном случае проводится с другими наследственными вариантами задержки роста, такими как синдром Рубинштейна-Тейби, Сильвера-Рассела, 3М и другими. Единственным достоверным способом постановки диагноза является проведение молекулярно-генетического исследования.

Причиной развития заболевания являются гетерозиготные нуклеотидные варианты в 33 и 34 экзонах гена SRCAP, которые приводят к образованию преждевременного стоп-кодона или сдвигу рамки считывания [6]. Данный ген кодирует связанную с SNF2 хроматин-ремоделирующую АТФ-азу, которая служит коактиватором для CREB-связывающего белка, более известного как CBP [7]. Механизм заболевания при СФХ связан с укорочением С-концевой части белка, содержащей ДНК-связывающие мотивы, что приводит к потере основной функции SRCAP активировать транскрипцию [6].

В настоящей работе приведены клинические и молекулярно-генетические характеристики 6 пациентов с синдромом Флоатинг-Харбор в Российской Федерации.

## ОПИСАНИЕ СЛУЧАЕВ

В исследование включено 6 пациентов с диагнозом «синдром Флоатинг-Харбор» (мальчики, n=4 и девочки, n=2), проходивших обследование в ФГБНУ «МГНЦ» и ФГБУ «НМИЦ эндокринологии» Минздрава России в период с 2017 по 2024 гг. Медиана возраста на момент обращения составила 2,9 года [ 1,7; 7,0 лет]. Основными жалобами при первичном обследовании являлись задержка роста и речевого развития. Ме роста на момент обследования составила -3,2 SD [ -3,9; -2,7], Ме ИМТ — -2,2 SD [ -3,2; -2,0] (табл. 1). В двух случаях диагностирована задержка внутриутробного развития (ЗВУР) (N1, N4), при этом все пациенты были от доношенной беременности, Ме 39,5 недели [ 38; 40 недель]. Из анамнеза жизни известно, что у всех пациентов отмечалась задержка речевого развития: первые слова после двух лет. В одном случае диагностирована задержка и моторного развития (N2): начало самостоятельной ходьбы с 2 лет 2 мес.

**Table:** Таблица 1. Клинические и молекулярно-генетические данные пациентов с синдромом Флоатинг-Харбор

N	1	2	3	4	5	6
Пол	муж	муж	жен	муж	муж	жен
Возраст на момент обращения	11 мес	8 лет	1,8 года	1,6 года	11,5 года	4,0 года
Роды, неделя	41	40	37	39	40	37
Длина, SD	-1,3	-0,4	N/A	-2,3	0,2	-0,5
Масса, SD	-2,8	-1,3	-1,1	-2,9	-1,1	-1,6
Окружность головы, SD	-6,9	-3,2	-1,1	-3,6	-0,7	N/A
Pост, SD	-5,7	-2,4	-2,6	-3,2	-3,1	-4,1
ИМТ, SD	3,4	-3,4	-2	-2,4	-3,6	-2
Характерные черты лица	+	+	+	+	+	+
Скелетные аномалии	Широкие кисти, стопы	Клинодактилия мизинцев	Закрытый эпифизиолиз бедренной кости	Лункообразные ногти на ногах	Клинодактилия мизинцев, вороонкообразная грудная клетка	Брахидактилия дистальной фаланги мизинцев
Речевое развитие	-	Слоги с 4 лет, короткие фразы с 8 лет	С задержкой, после 2 лет	С задержкой, после 2 лет	Первые слова в 2 года	Речь отсутствует
Моторное развитие	Грубая задержка	Сидит с 9 мес, ходит с 2 лет 2 мес	С задержкой	Ползает с 10 мес, ходит с 1 года 4 мес	Ходит с 1 года 4 мес	Ходит с 1,5 лет
Другие фенотипические особенности	Двусторонний крипторхизм, скрытый половой член	Пролапс митрального клапана	Пупочная грыжа	-	-	-
Наследственность	не отягощена	не отягощена	не отягощена	не отягощена	не отягощена	не отягощена
Вариант в SRCAP	c.7330C>T; p.Arg2444*	c.7330C>T; p.Arg2444*	c.7285dup; p.Cys2429Leufs*14	c.7274dup; p.Pro2426Thrfs*17	c.7917_7924del; p.Glu2640Alafs*6	c.7330C>T; p.Arg2444*

При осмотре у всех обследуемых обращал на себя внимание ряд лицевых фенотипических особенностей: треугольное лицо, короткий фильтр, тонкая верхняя губа, нос с тонким основание и широким кончиком, низко посаженные большие уши. Микроцефалия выявлена в трех случаях: N1, N2, N4. Костные аномалии выявлены в четырех случаях: клинодактилия мизинцев выявлена в двух случаях (N2, N5), брахидактилия в одном (N7) и закрытый эпифизиолиз бедренной кости так же в одном случае (N4). К другим аномалиям развития относились: пролапс митрального клапана (N2), двусторонний крипторхизм (N1) и пупочная грыжа (N3). Семейный анамнез не был отягощен ни в одном из случаев.

Молекулярно-генетическое исследование выполнено методом полноэкзомного секвенирования. У 6 обследованных пациентов выявлено 4 различных нуклеотидных варианта в гене SRCAP (NM_006662.3) (рис. 1), все в гетерозиготном состоянии: в трех случаях (N1, N2, N6) c.7330C>T (p.Arg2444*), в остальных — варианты c.7285dup (p.Cys2429Leufs*14) (N3), c.7274dup (p.Pro2426Thrfs*17) (N4), c.7917_7924del, (p.Glu2640Alafs*6) (N5). Кровь родителей была доступна в 4 случаях (N1–4), аналогичных изменений нуклеотидной последовательности выявлено не было, что указывало на происхождение вариантов de novo.

**Figure:**
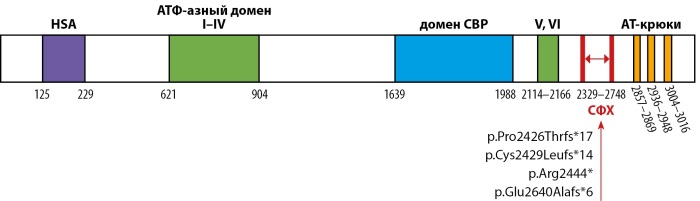
Рисунок 1. Схематическое изображение белка SRCAP с указанием аминокислотных границ доменов и области, ассоциированной с синдромом Флоатинг-Харбор; нуклеотидные изменения, выявленные у пациентов в настоящем исследовании.

## ОБСУЖДЕНИЕ

Свое название синдром Флоатинг-Харбор получил благодаря двум больницам Boston Floating Hospital и Harbor General Hospital, в которых описаны два первых наблюдения заболевания [2][8]. Отличительными чертами данного синдрома является лицевой дисморфизм, задержка роста, скелетные аномалии и задержка речевого развития. Черепно-лицевые аномалии включают треугольную форму лица, глубоко посаженные глаза с длинными ресницами, тонкую верхнюю губу с красной каймой, макростомию, нос с узкой переносицей, широким основанием и кончиком, выступающую калумеллу, короткий фильтр, низко посаженные уши [3]. Скелетные аномалии затрагивают в основном область верхних и нижних конечностей, возможны такие изменения, как брахидактилия, клинодактилия, широкие кончики пальцев («барабанные палочки»), дисплазия тазобедренных суставов, односторонняя или двусторонняя деформация ключиц [3]. Задержка речевого развития является важной отличительной чертой заболевания, у ряда пациентов также была диагностирована легкая умственная отсталость и синдром дефицита внимания [3].

Антропометрические показатели пациентов с синдромом Флоатинг-Харбор оценены в исследовании Nikkel с соавт. (n=49): конечный рост женщин варьировал от -4 SD до -2 SD, мужчин — -4,5 SD – -2 SD; ИМТ имел выраженную вариабельность, что не позволило определить характерные для заболевания показатели. В 26% случаев диагностирована ЗВУР, микроцефалия выявлена в 14% [3]. На сегодняшний день остается открытым вопрос о причине формирования низкорослости, существуют единичные публикации о диагностике СТГ-дефицита у данных пациентов [9][10]. Между тем делаются попытки лечения гормоном роста, окончательные данные об эффективности которого пока отсутствуют. Bo с соавт. проанализировали литературные данные с результатом лечения 22 пациентов с СФХ. На фоне терапии, длительность которой составляла от 1 до 9 лет (медиана: 3,2 года), ∆SDS роста варьировал от -0,4 до 2,1 (медиана: 1,2), при этом никто из пациентов не достиг конечного роста [11].

Помимо основных клинических проявлений синдрома, в различных исследования у пациентов диагностированы нарушения со стороны офтальмологии (косоглазие, дальнозоркость, нистагм), оториноларингологии (рецидивирующий средний отит, кохлеарная аномалия, дефекты твердого и мягкого неба, атрезия хоан), стоматологии (маленькие зубы, кариес), сердечно-сосудистой системы (пороки развития сердца), ЖКТ (целиакия, грыжи) и мочеполовой системы (крипторхизм, аномалии почечной системы) [3]. Частота данных проявления не позволяет включить их в основной симптомокомплекс, однако требует более тщательного обследования пациентов с СФХ.

Молекулярная основа синдрома Флоатинг-Харбор была определена в 2012 году. В работе Hood с соавт. у 5 неродственных пробандов с фенотипом заболевания проведен поиск редких вариантов нуклеотидной последовательности методом секвенирования экзома, и во всех случаях идентифицированы изменения в экзоне 34 гена SRCAP [6]. Важно отметить, что все выявленные варианты приводили к укорочению длины белка (нонсенс-мутации или варианты со сдвигом рамки считывания) и были сгруппированы между кодонами 2407 и 2517. Позднее, благодаря новым исследованиям, ассоциированная область была расширена, и сейчас располагается между кодонами 2329 и 2748 (http://www.hgmd.org) (рис. 1). Позднее было обнаружено, что миссенс-варианты в гене SRCAP ассоциированы с болезнью Альцгеймера — с поздним началом и, реже, психиатрическими нарушениями, такими как расстройство аутистического спектра [12][13]. В то же время приводящие к синтезу укороченного белка варианты, рассеянные на всем протяжении гена строго проксимально и дистально относительно локуса СФХ, ассоциированы с нарушением развития нервной системы, не имеющим каких-либо специфических признаков, которое характеризуется умственной отсталостью в сочетании с психиатрической симптоматикой и в настоящее время идентифицируется как «не-СФХ нарушение развития нервной системы» [14].

Ген SRCAP (SNF2-related CBP activator protein) расположен на коротком плече хромосомы 16 (16p11.2) и кодирует одноимённый белок, обладающий АТФ-азной активностью и являющийся одним из основных компонентов мультипротеинового комплекса ремоделирования хроматина SRCAP-С [7].

Белок SRCAP как функциональная единица впервые выявлен в ходе исследования взаимодействий N-концевого домена активации транскрипции белка CBP [15]. Более детальный анализ аминокислотной последовательности обнаруженного белка показал наличие AТФ-азного домена, который оказался высоко гомологичен аналогичному домену белков семейства ремоделлеров хроматина Snf2. Доменная структура белка SRCAP включает N-концевой домен HSA (Helicase-SANT-associated), домен взаимодействия с CBP и три С-концевых «АТ-крюка» [6]. АТ-крюки приставляют из себя высококонсервативные положительно заряженные палиндромные мотивы Аргинин-Глицин-Аргинин, которые, предположительно, отвечают за связывание с AT-богатыми участками ДНК посредством контактов с малой бороздкой двойной спирали [16].

Основной функцией белка SRCAP считается его участие во включении в нуклеосомы димеров гистонов H2A.Z-H2B. H2A.Z — консервативный вариант гистона H2A, играющий важную роль в процессах ремоделирования хроматина для репарации ДНК и регуляции транскрипционной активности, а также в репликации ДНК и стабилизации состояния хроматина при расхождении хромосом [17]. Истощение H2A.Z приводит к глобальному изменению регуляции транскрипции в клетках.

Комплекс SRCAP-С также является важным звеном в CBP-опосредованной активации транскрипции, будучи непосредственным коактиватором CBP [15]. Белок СBP и его гомолог p300 являются ацетилтрансферазами, имеющими широкую сеть взаимодействия с различными транскрипционными факторами и регуляторными белками. CBP/p300 участвуют в активации цАМФ-опосредованной транскрипции, напрямую ремоделируют хроматин, благодаря способности ацетилировать, в частности гистоновые белки, а также активируют транскрипцию как напрямую, так и через кофакторы, физически связывая и стабилизируя белки в комплексах инициации транскрипции [18]. Авторы также установили выраженный доминантно-негативный эффект с подавлением активаторной функции при использовании фрагмента SRCAP, имеющего только домен взаимодействия с СBP без дистальных ДНК-связывающих мотивов, что может иметь значение при сопоставлении этих данных с известным спектром патогенных вариантов, выявляемых у пациентов с CФХ. Функциональная связь с CBP/p300 интересна также в плане некоторого фенотипического сходства пациентов с СФХ и синдромом Рубинштейна-Тейби, за развитие которого ответственны дефекты в генах, кодирующих эти белки [18].

В настоящее время описано около ста патогенных и вероятно патогенных вариантов в гене SRCAP, но с клиническим фенотипом СФХ ассоциированы примерно 30 из них. Выделяют два часто повторяющихся среди пациентов с СФХ варианта — c.7330C>T p.Arg2444* и c.7303C>T, p.Arg2435*, определяющих «горячие точки» в экзоне 34. Все описанные варианты приводят к формированию преждевременного стоп-кодона вследствие однонуклеотидных замен или небольших делеций и инсерций со сдвигом рамки считывания. Таким образом, во всех случаях предполагается потеря ДНК-связывающих мотивов, расположенных в дистальной части гена, и нарушение взаимодействия ДНК с комплексами регуляторных белков, вследствие чего по доминантно-негативному механизму субстрат, связываемый укороченным белком с нарушенной функцией, блокируется от взаимодействия с белком дикого типа. Экспериментальных доказательств этого механизма на настоящий момент не получено, однако обсуждается также вероятность изменения локализации укороченного белка с ядерной на цитоплазматическую, что было выявлено при исследовании эмбриональных клеток нервного гребня человека [7].

Все нуклеотидные изменения, выявленные в ходе настоящего исследования, также расположены в области между кодонами 2329 и 2748 и приводят к укорочению длины белка. Два из выявленных изменения в гене SRCAP ранее описаны как патогенные: c.7330C>T p.Arg2444* и c.7274dup p.Pro2426Thrfs*17, вероятно, также отмечая горячие точки. Два не описанных ранее варианта c.7285dup p.Cys2429Leufs*14 и c.7917_7924del p.Glu2640Alafs*6 также приводят к укорочению белкового продукта и в соответствии с критериями, используемыми для интерпретации результатов секвенирования, оценены как вероятно патогенные [19][20]. Клинически у пациентов диагностирован классический фенотип синдрома Флоатинг-Харбор. Интересно, что в случае N2 зарегистрирована также задержка моторного развития, о чем ранее не сообщалось. Однако учитывая, что это единичный случай, нельзя исключить другие причины формирования данного состояния.

## ЗАКЛЮЧЕНИЕ

Впервые для Российской Федерации описана серия клинических случаев синдрома Флоатинг-Харбор. Полученные нами данные указывают на необходимость проведения дифференциального диагноза СФХ с другими наследственными синдромальными формами низкорослости. Проведение молекулярно-генетического исследования является единственным способом установки патогенетического диагноза. Полученные результаты важны для дальнейшего медико-генетического консультирования семьи и решения вопроса о тактике наблюдения и ведения пациента.

## ДОПОЛНИТЕЛЬНАЯ ИНФОРМАЦИЯ

Источники финансирования. Работа выполнена в рамках государственного задания Минобрнауки России для ФГБНУ «МГНЦ».

Конфликт интересов. Авторы декларируют отсутствие явных и потенциальных конфликтов интересов, связанных с содержанием настоящей статьи.

Участие авторов. Все авторы одобрили финальную версию статьи перед публикацией, выразили согласие нести ответственность за все аспекты работы, подразумевающую надлежащее изучение и решение вопросов, связанных с точностью или добросовестностью любой части работы.

Согласие пациента. Добровольное информированное согласие законных представителей пациенов на публикацию в журнале “Проблемы эндокринологии” получены.

## References

[cit1] Lacombe D., Patton M. A., Elleau C., Battin J. (2005). Floating-Harbor syndrome: description of a further patient, review of the literature, and suggestion of autosomal dominant inheritance. European Journal of Pediatrics.

[cit2] Pelletier G, Feingold M. Case report 1. Syndrome Identification J. 1973;1:8–9

[cit3] Nikkel Sarah M, Dauber Andrew, de Munnik Sonja, Connolly Meghan, Hood Rebecca L, Caluseriu Oana, Hurst Jane, Kini Usha, Nowaczyk Malgorzata J M, Afenjar Alexandra, Albrecht Beate, Allanson Judith E, Balestri Paolo, Ben-Omran Tawfeg, Brancati Francesco, Cordeiro Isabel, da Cunha Bruna Santos, Delaney Louisa A, Destrée Anne, Fitzpatrick David, Forzano Francesca, Ghali Neeti, Gillies Greta, Harwood Katerina, Hendriks Yvonne M C, Héron Delphine, Hoischen Alexander, Honey Engela Magdalena, Hoefsloot Lies H, Ibrahim Jennifer, Jacob Claire M, Kant Sarina G, Kim Chong Ae, Kirk Edwin P, Knoers Nine V A M, Lacombe Didier, Lee Chung, Lo Ivan F M, Lucas Luiza S, Mari Francesca, Mericq Veronica, Moilanen Jukka S, Møller Sanne Traasdahl, Moortgat Stephanie, Pilz Daniela T, Pope Kate, Price Susan, Renieri Alessandra, Sá Joaquim, Schoots Jeroen, Silveira Elizabeth L, Simon Marleen E H, Slavotinek Anne, Temple I Karen, van der Burgt Ineke, de Vries Bert B A, Weisfeld-Adams James D, Whiteford Margo L, Wierczorek Dagmar, Wit Jan M, Yee Connie Fung On, Beaulieu Chandree L, White Sue M, Bulman Dennis E, Bongers Ernie, Brunner Han, Feingold Murray, Boycott Kym M (2013). The phenotype of Floating-Harbor syndrome: clinical characterization of 52 individuals with mutations in exon 34 of SRCAP. Orphanet Journal of Rare Diseases.

[cit4] Seifert Wenke, Meinecke Peter, Krüger Gabriele, Rossier Eva, Heinritz Wolfram, Wüsthof Achim, Horn Denise (2014). Expanded spectrum of exon 33 and 34 mutations in SRCAP and follow-up in patients with Floating-Harbor syndrome. BMC Medical Genetics.

[cit5] Kehrer M., Beckmann A., Wyduba J., Finckh U., Dufke A., Gaiser U., Tzschach A. (2013). Floating‐Harbor syndrome: SRCAP mutations are not restricted to exon 34. Clinical Genetics.

[cit6] Hood Rebecca L., Lines Matthew A., Nikkel Sarah M., Schwartzentruber Jeremy, Beaulieu Chandree, Nowaczyk Małgorzata J.M., Allanson Judith, Kim Chong Ae, Wieczorek Dagmar, Moilanen Jukka S., Lacombe Didier, Gillessen-Kaesbach Gabriele, Whiteford Margo L., Quaio Caio Robledo D.C., Gomy Israel, Bertola Debora R., Albrecht Beate, Platzer Konrad, McGillivray George, Zou Ruobing, McLeod D. Ross, Chudley Albert E., Chodirker Bernard N., Marcadier Janet, Majewski Jacek, Bulman Dennis E., White Susan M., Boycott Kym M. (2012). Mutations in SRCAP, Encoding SNF2-Related CREBBP Activator Protein, Cause Floating-Harbor Syndrome. The American Journal of Human Genetics.

[cit7] Messina Giovanni, Prozzillo Yuri, Delle Monache Francesca, Santopietro Maria Virginia, Atterrato Maria Teresa, Dimitri Patrizio (2021). The ATPase SRCAP is associated with the mitotic apparatus, uncovering novel molecular aspects of Floating-Harbor syndrome. BMC Biology.

[cit8] Leisti J, Hollister DW, Rimoin DL. The Floating-Harbor syndrome. Birth Defects Orig Artic Ser. 1975;11(5):305 1218224

[cit9] Galli-Tsinopoulou Assimina, Kyrgios Ioannis, Emmanouilidou Eleftheria, Maggana Ioanna, Kotanidou Eleni, Kokka Paraskevi, Stylianou Charilaos (2014). Growth Hormone Deficiency: an unusual presentation of Floating Harbor Syndrome. Hormones.

[cit10] Homma Thais K., Freire Bruna L., Honjo Rachel, Dauber Andrew, Funari Mariana F.A., Lerario Antonio M., Albuquerque Edoarda V.A., Vasques Gabriela A., Bertola Debora R., Kim Chong A., Malaquias Alexsandra C., Jorge Alexander A.L. (2019). Growth and Clinical Characteristics of Children with Floating-Harbor Syndrome: Analysis of Current Original Data and a Review of the Literature. Hormone Research in Paediatrics.

[cit11] Bo Hui, Jiang Lihong, Zheng Jiaqi, Sun Jie (2021). Floating-Harbor Syndrome Treated With Recombinant Human Growth Hormone: A Case Report and Literature Review. Frontiers in Pediatrics.

[cit12] Vardarajan Badri N., Tosto Giuseppe, Lefort Roger, Yu Lei, Bennett David A., De Jager Philip L., Barral Sandra, Reyes-Dumeyer Dolly, Nagy Peter L., Lee Joseph H., Cheng Rong, Medrano Martin, Lantigua Rafael, Rogaeva Ekaterina, St George-Hyslop Peter, Mayeux Richard (2017). Ultra-rare mutations in SRCAP segregate in Caribbean Hispanic families with Alzheimer disease. Neurology Genetics.

[cit13] Iossifov Ivan, O’Roak Brian J., Sanders Stephan J., Ronemus Michael, Krumm Niklas, Levy Dan, Stessman Holly A., Witherspoon Kali T., Vives Laura, Patterson Karynne E., Smith Joshua D., Paeper Bryan, Nickerson Deborah A., Dea Jeanselle, Dong Shan, Gonzalez Luis E., Mandell Jeffrey D., Mane Shrikant M., Murtha Michael T., Sullivan Catherine A., Walker Michael F., Waqar Zainulabedin, Wei Liping, Willsey A. Jeremy, Yamrom Boris, Lee Yoon-ha, Grabowska Ewa, Dalkic Ertugrul, Wang Zihua, Marks Steven, Andrews Peter, Leotta Anthony, Kendall Jude, Hakker Inessa, Rosenbaum Julie, Ma Beicong, Rodgers Linda, Troge Jennifer, Narzisi Giuseppe, Yoon Seungtai, Schatz Michael C., Ye Kenny, McCombie W. Richard, Shendure Jay, Eichler Evan E., State Matthew W., Wigler Michael (2014). The contribution of de novo coding mutations to autism spectrum disorder. Nature.

[cit14] Rots Dmitrijs, Chater-Diehl Eric, Dingemans Alexander J.M., Goodman Sarah J., Siu Michelle T., Cytrynbaum Cheryl, Choufani Sanaa, Hoang Ny, Walker Susan, Awamleh Zain, Charkow Joshua, Meyn Stephen, Pfundt Rolph, Rinne Tuula, Gardeitchik Thatjana, de Vries Bert B.A., Deden A. Chantal, Leenders Erika, Kwint Michael, Stumpel Constance T.R.M., Stevens Servi J.C., Vermeulen Jeroen R., van Harssel Jeske V.T., Bosch Danielle G.M., van Gassen Koen L.I., van Binsbergen Ellen, de Geus Christa M., Brackel Hein, Hempel Maja, Lessel Davor, Denecke Jonas, Slavotinek Anne, Strober Jonathan, Crunk Amy, Folk Leandra, Wentzensen Ingrid M., Yang Hui, Zou Fanggeng, Millan Francisca, Person Richard, Xie Yili, Liu Shuxi, Ousager Lilian B., Larsen Martin, Schultz-Rogers Laura, Morava Eva, Klee Eric W., Berry Ian R., Campbell Jennifer, Lindstrom Kristin, Pruniski Brianna, Neumeyer Ann M., Radley Jessica A., Phornphutkul Chanika, Schmidt Berkley, Wilson William G., Õunap Katrin, Reinson Karit, Pajusalu Sander, van Haeringen Arie, Ruivenkamp Claudia, Cuperus Roos, Santos-Simarro Fernando, Palomares-Bralo María, Pacio-Míguez Marta, Ritter Alyssa, Bhoj Elizabeth, Tønne Elin, Tveten Kristian, Cappuccio Gerarda, Brunetti-Pierri Nicola, Rowe Leah, Bunn Jason, Saenz Margarita, Platzer Konrad, Mertens Mareike, Caluseriu Oana, Nowaczyk Małgorzata J.M., Cohn Ronald D., Kannu Peter, Alkhunaizi Ebba, Chitayat David, Scherer Stephen W., Brunner Han G., Vissers Lisenka E.L.M., Kleefstra Tjitske, Koolen David A., Weksberg Rosanna (2021). Truncating SRCAP variants outside the Floating-Harbor syndrome locus cause a distinct neurodevelopmental disorder with a specific DNA methylation signature. The American Journal of Human Genetics.

[cit15] Johnston Holly, Kneer Joni, Chackalaparampil Isaac, Yaciuk Peter, Chrivia John (2002). Identification of a Novel SNF2/SWI2 Protein Family Member, SRCAP, Which Interacts with CREB-binding Protein. Journal of Biological Chemistry.

[cit16] Zhao Jianfei, Favero David S, Qiu Jiwen, Roalson Eric H, Neff Michael M (2014). Insights into the evolution and diversification of the AT-hook Motif Nuclear Localized gene family in land plants. BMC Plant Biology.

[cit17] Yu Jiali, Sui Fengrui, Gu Feng, Li Wanjun, Yu Zishuo, Wang Qianmin, He Shuang, Wang Li, Xu Yanhui (2024). Structural insights into histone exchange by human SRCAP complex. Cell Discovery.

[cit18] Park Elizabeth, Kim Yunha, Ryu Hyun, Kowall Neil W., Lee Junghee, Ryu Hoon (2013). Epigenetic Mechanisms of Rubinstein–Taybi Syndrome. NeuroMolecular Medicine.

[cit19] Richards Sue, Aziz Nazneen, Bale Sherri, Bick David, Das Soma, Gastier-Foster Julie, Grody Wayne W., Hegde Madhuri, Lyon Elaine, Spector Elaine, Voelkerding Karl, Rehm Heidi L. (2015). Standards and guidelines for the interpretation of sequence variants: a joint consensus recommendation of the American College of Medical Genetics and Genomics and the Association for Molecular Pathology. Genetics in Medicine.

[cit20] Рыжкова О.П., Кардымон О.Л., Прохорчук Е.Б., Коновалов Ф.А., Масленников А.Б., Степанов В.А., Афанасьев А.А., Заклязьминская Е.В., Ребриков Д.В., Савостьянов К.В., Глотов А.С., Костарева А.А., Павлов А.Е., Голубенко М.В., Поляков А.В., Куцев С.И. (2020). Руководство по интерпретации данных последовательности ДНК человека, полученных методами массового параллельного секвенирования (MPS) (редакция 2018, версия 2). Nauchno-prakticheskii zhurnal «Medicinskaia genetika».

